# Musculoskeletal Anatomy Education: A Survey of North American Medical Programs

**DOI:** 10.1002/ca.24282

**Published:** 2025-04-28

**Authors:** Taylor Orchard, Jason Peeler

**Affiliations:** ^1^ Max Rady College of Medicine, University of Manitoba Winnipeg Canada

**Keywords:** allopathic medical program, human anatomy, orthopedics, preclinical education, undergraduate medical education

## Abstract

Postgraduate data reveal that most physicians lack adequate anatomical knowledge and clinical confidence when practicing musculoskeletal (MSK) medicine. Curricular data from nationally accredited medical programs clearly indicate that the total time dedicated to gross anatomy instruction has decreased over recent decades. However, little information is available regarding the MSK anatomy learning environment across accredited medical programs in North America. The purpose of this study was to document the current state of preclinical MSK anatomy education across North American medical programs. A survey was sent to all 14 English‐speaking Canadian and 135 of the 158 American accredited medical programs. The survey had a 100% response rate from Canadian programs and 43% from American programs. The results indicated that the mean time spent learning preclinical MSK anatomy varied widely across both Canadian (29.8 h ± 13.7, range 12–60, median 29, mode 12) and American (50.8 h ± 46.2, range 2–280, median 35, mode 30) programs, most of them integrating anatomy learning into the clinical learning environment (56%). All but one program reported using cadaveric‐based instruction (99%) and the majority taught radiological correlates (94%) and surface anatomy (71%) within their curriculum. Diverse modes of instruction were used by all programs, but didactic lectures remained the most frequent form (89%). While a variety of learning resources were used to support student learning, the type of resource varied significantly, Canadian programs most commonly providing a “curriculum‐specific” notes package (86%) and American programs most commonly requiring an anatomy atlas (84%). Summative and formative methods of evaluation were used by most programs (96%), final written examinations (79%) and ongoing in‐course evaluation (81%) being most popular. The results serve to document the current state of preclinical MSK anatomy education within nationally accredited allopathic medical programs and to illustrate the wide variability of the learning environment. Future research should be directed at establishing consistent standards for preclinical MSK anatomy education and investigating the long‐term effects on knowledge retention and clinical confidence.

## 1 Introduction

Musculoskeletal (MSK) injuries and pathologies are the leading cause of disabilities globally, accounting for just over 20% of all emergency room visits, and are among the most common reasons a patient consults a physician (Fontánez et al. 2021; Weinstein et al. 2014). The estimated annual cost of providing MSK care to North Americans is thought to exceed $900 billion (Weinstein et al. 2014; Anderson and Althausen 2016), with demand increasing as the life expectancy of the aging population rises (Kontis et al. 2017). While a clear understanding and ability to apply anatomical knowledge in a clinically relevant manner is an essential element of physician training (Boyer 2005; Day and Ahn 2010; McBride and Drake 2018), research indicates that a very small percentage (~3%) of preclinical curricular time is dedicated to MSK education (Pinney and Regan 2001; Harkins et al. 2023; Chow et al. 2021). Additionally, many physicians lack adequate anatomical knowledge and confidence when practicing MSK medicine (Day and Ahn 2010; DiGiovanni et al. 2014, 2012; Grunfeld et al. 2012), and it has been reported that many medical students feel their training in MSK‐related topics prepares them inadequately for clinical practice (Sabesan et al. 2018).

Evidence clearly indicates that the total time dedicated to human anatomy instruction across nationally accredited medical programs in both the United States and Canada has decreased dramatically during recent decades (McBride and Drake 2018; Drake et al. 2009; Rockarts et al. 2020). However, little information is available about the effect of these global changes in human anatomy curricula on the MSK anatomy learning environment across programs. During the last decade, only a handful of articles have been published on curricular topics related to MSK anatomy education within accredited medical programs, and most of these publications reported either curricular data specific to their home institutions or curricular topics specific to the postgraduate MSK anatomy training environment (DiGiovanni et al. 2014; Sabesan et al. 2018; Peeler 2022, 2024; Peeler et al. 2018; Whelan et al. 2018; Orchard et al. 2024; Lisk et al. 2014; Hankin et al. 2023; Harmon et al. 2024). Despite efforts by organizations such as the International Federation of Associations of Anatomists (IFAA), who have published recommendations for core MSK anatomy syllabi in medical education (Tubbs et al. 2014; Webb et al. 2019), there remains a paucity of data describing the preclinical MSK anatomy learning environment within nationally accredited allopathic medical programs in North America. Wang et al. (2021) is currently the only recent publication to document and compare the MSK anatomy curricula among many medical programs, all of which were California‐based (Wang et al. 2021). As such, there is a lack of data to help guide the development and implementation of MSK anatomy programming.

The purpose of this investigation was to document the current state of preclinical MSK anatomy education across nationally accredited medical programs in North America. The goals of this study were to: (1) Address the current significant knowledge gap regarding preclinical MSK anatomy education within nationally accredited Canadian and American medical programs; and (2) Identify curricular/pedagogical “norms” in preclinical MSK anatomy education. It was hypothesized that nationally accredited AAMC (Association of American Medical Colleges) and AFMC (Association of Faculties of Medicine of Canada) allopathic medical programs would display curricular homogeneity in providing MSK anatomy education to medical students during their preclinical years of training.

## 2 Materials and Methods

A cross‐sectional survey design, adopted from the previous work of Wang et al. (2021) and Peeler et al. (2024), was used for this investigation (Wang et al. 2021; Peeler et al. 2024). Following institutional ethics board approval (Ethics #: HS25881 (H2023:060)), a letter of introduction that included the main purpose and objectives of the investigation and a web link to an electronic MSK anatomy survey (hosted through SurveyMonkey) was emailed to all nationally accredited Canadian undergraduate allopathic medical programs with a request for a single institutional response. A survey consisting of 11 questions and eight follow‐up questions was emailed to all 14 English‐speaking Canadian medical programs during the fall of 2022. During the fall of 2023, the same survey (inclusive of all 19 questions) was sent to 135 of the 158 American medical programs. Contact information for each institution was accessed through publicly available websites and with the support of the American Association for Anatomy (AAA), the American Association of Clinical Anatomists (AACA), and the Canadian Orthopedic Association (COA). Unfortunately, contact information for 23 American programs could not be accessed. Participation in this survey‐based investigation was completely optional for all programs, and no incentive was provided.

Data organization and statistical analysis were completed using Microsoft Excel software (*Microsoft Office Professional Plus 2016, Santa Rosa, California*) and Statistics Kingdom: Website for Statistical Computation (https://www.statskingdom.com). Aggregate data were summarized using descriptive statistics, while continuous data were described using means, standard deviations, medians, modes, and minimum/maximum values. Independent t‐tests and Mann–Whitney U Rank tests were used to compare the Canadian and American data. Spearman Rank correlation testing was used to examine the relationship between key outcome measures. Results were considered statistically significant if *p* ≤ 0.05.

## 3 Results

Complete survey responses were received from all 14 English‐speaking Canadian medical schools (100% response rate) and 58 American medical programs (43% response rate).

Curricular data (see Table 1) indicated that Canadian programs provided an average of 29.8 h (Std ± 13.7, (range 12–60), Mdn = 29, Mo = 12) of preclinical MSK anatomy instruction, while American programs provided an average of 50.8 h (Std ± 46.1 (range 2–280), Mdn = 35, Mo = 30). Independent t‐tests indicated no significant differences in curricular hours between the Canadian and American programs (*p* = 0.10). Among the Canadian programs, eight (57%) reported using an integrated approach to teaching in which MSK anatomy content was delivered in conjunction with clinical topics; four (29%) delivered anatomy as a standalone course; only two (14%) delivered their content as part of a larger systems‐based human anatomy course. Similarly, 32 American programs (55%) used an integrated approach to delivery; six (10%) delivered a stand‐alone MSK anatomy curriculum; and 20 (35%) used a large systems‐based human anatomy course. Mann–Whitney U Rank testing indicated no significant differences in the modes of delivery used by Canadian and American programs (*p* = 0.69). Data also indicated that eleven Canadian (79%) and 40 American (71%) programs included surface anatomy/palpation as part of their curricula, and 13 Canadian (93%) and 55 American (98%) programs included radiological correlates (X‐ray, CT, MRI) in their MSK anatomy curriculum. Mann–Whitney U Rank testing illustrated no significant differences between Canadian and American results (surface anatomy/palpation: *p* = 0.60; radiological correlates: *p* = 0.30).

**TABLE 1:** MSK anatomy learning environment by nationally accredited medical programs.

		AFMC (*n* = 14)	AAMC (*n* = 58)	Total (*n* = 72)
Response rate		100% (14)	43% (58)	48% (72)
Curricular time (hours)	Mean ± std (range)	29.8 ± 13.7 (12–60)	50.8 ± 46.1 (2–280)	46.6 ± 42.5 (2–280)
	Median (mode)	29 (12)	35 (30)	34 (30)
Stand‐alone curriculum		29% (4)	10% (6)	14% (10)
Systems‐based curriculum		14% (2)	35% (20)	31% (22)
Integrated curriculum		57% (8)	55% (32)	56% (40)
Surface anatomy		79% (11)	71% (40)^a^	71% (51)
Radiological correlates		93% (13)	98% (55)^a^	94% (68)

^a^ Denotes variables that had an *n* = 56 where two programs failed to complete the question on the survey. (*p ≤ 0.05).

Data also indicated that most Canadian (13%–93%) and American (58%–100%) programs used cadaveric specimens to support MSK anatomy learning. Among the Canadian programs, only one (7%) reported using a strictly dissection‐based curriculum; six (43%) used prosection‐based; while six (43%) reported incorporating a combination of dissection and prosection into their curricula. Surprisingly, one Canadian program (7%) did not use cadaveric specimens to teach MSK anatomy. Comparatively, 12 American programs (21%) used dissection‐based labs; 11 (19%) used prosection‐based; and 35 (60%) used a combination of dissection and prosection. Mann–Whitney U Rank tests indicated no significant difference between the Canadian and American programs in the types of cadaveric‐based instruction used (*p* = 0.71). Data also indicated that Canadian programs dedicated an average of 14.8 h (±5.7, range: 8–20) to dissection‐based instruction and an average of 7.1 h (±4.1, range: 2–12) to prosection‐based teaching. In contrast, American programs devoted an average of 23.9 h (±25.5, range: 3–160) to dissection‐based learning and 10.7 h (±26.8, range: 0.2–180) to prosection‐based learning. Interestingly, a large percentage of programs (ten Canadian—71.4%, 41 American—70.7%) reported incorporating other learning aids (e.g., muscle models, visualization software, etc.) into their labs. The survey results also revealed that approximately half of the programs (seven Canadian—50%; 31 American—53%) delivered their curricula exclusively during the first year of medical training; sixteen (two Canadian—14%; 14 American—24%) during the second year; and eight (two Canadian—25%; six American—10%) across both the first and second years.

**FIGURE 1:**
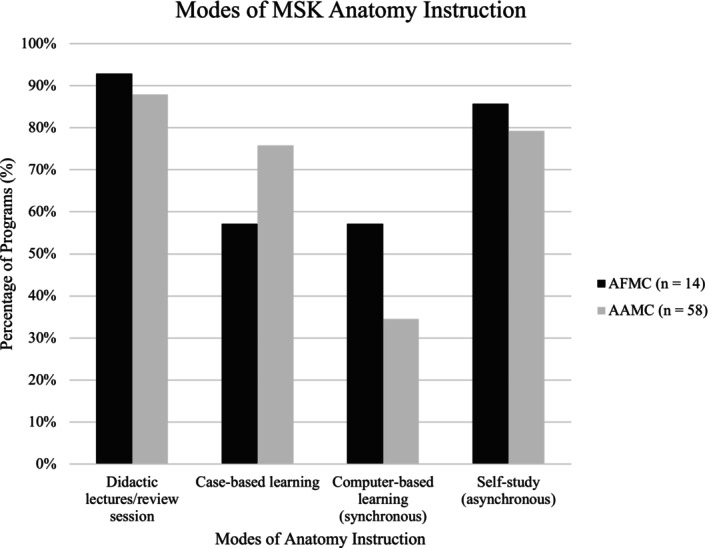
Modes of MSK anatomy instruction. Modes used to deliver MSK anatomy curricula across AFMC and AAMC accredited medical programs. (*p ≤ 0.05).

**FIGURE 2:**
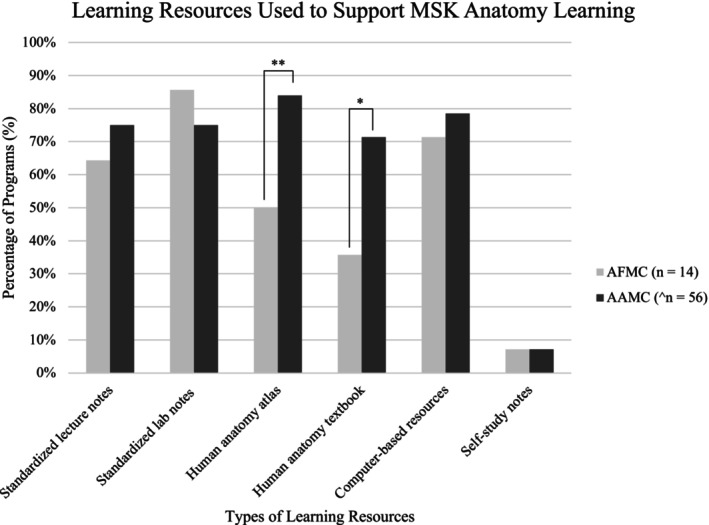
Learning resources used to support MSK anatomy learning. Learning resources used by AFMC and AAMC accredited medical programs during MSK anatomy education. Two American medical programs were excluded from this calculation owing to incomplete responses to this question (*n* = 56). (*p ≤ 0.05, **p ≤ 0.01).

In addition to cadaveric‐based learning, programs also used a host of different modes of instruction to support MSK anatomy learning (Figure 1). Among the most popular methods were didactic lectures (13 Canadian—93%, 51 American—88%), and self‐study/asynchronous learning (12 Canadian—86%, 46 American—79%). From a learning resource standpoint, the most commonly used resources among Canadian programs were institution‐specific notes packages (12%–86%) and computer‐based resources (10%–71%). In contrast, the most common resources used by American programs were an anatomy atlas (47%–84%) and computer‐based resources (44%–79%). Mann–Whitney U Rank tests indicated a significant difference between the percentages of Canadian and American programs that required the use of an anatomy atlas/textbook (*p* = 0.000001228; *p* = 0.03). Surprisingly, only one Canadian (7%) and four American (7%) programs still required students to develop their own study notes (Figure 2). Survey data also indicated significant differences between programs in what was considered a core or must‐know topic in MSK anatomy (only 4/17 topics were taught by all Canadian programs, and 0/17 topics were taught by all American programs). There was also little consistency in the extent to which each program covered the core topics (see Table 2). Despite this, when respondents were asked to use a 5‐point Likert scale (1 = poorly prepared, 5 = extremely prepared) to assess how prepared students were to apply their MSK anatomy knowledge in a clinically relevant/confident manner, all Canadian (100%) and the vast majority of American (94%) programs believed that their students were adequately prepared (Canadian 3.25/5; American 3.65/5). There was no correlation in the relationship between the topics covered and the level of student preparedness. Beyond this, there was no relationship between student preparedness and the number of hours included in the programs' MSK anatomy curricula.

**TABLE 2:** Core topics covered by nationally accredited medical programs.

	Canadian (*n* = 8)^a^		American (*n* = 57)		*p*
	% of programs	Mean rating (/4)	% of programs	Mean rating (/4)	
Embryology of MSK system	13% (1)	1.38 ± 1.19	60% (34)	2.42 ± 1.12	0.01*
Histology of the MSK system	25% (2)	1.75 ± 1.04	79% (45)	2.96 ± 0.84	0.002**
Anatomical terminology	75% (6)	3.13 ± 1.13	91% (52)	3.33 ± 0.74	0.18
Bones (axial & appendicular skeleton)	100% (8)	3.63 ± 0.52	89% (51)	3.28 ± 0.82	0.35
Bony features/vocabulary	100% (8)	3.38 ± 0.52	74% (42)	3.05 ± 0.91	0.10
Joints (classifications & architecture)	88% (7)	3.25 ± 0.71	67% (38)	2.81 ± 0.93	0.24
Joint function & range of motion	88% (7)	2.75 ± 1.17	72% (41)	2.86 ± 0.83	0.36
Ligaments & supporting structures	63% (5)	2.88 ± 0.83	74% (42)	2.91 ± 0.83	0.52
Ligamentous attachments	13% (1)	1.38 ± 1.41	47% (27)	2.37 ± 0.90	0.07
Muscles	88% (7)	3.38 ± 0.74	96% (55)	3.54 ± 0.57	0.27
Muscular attachments	38% (3)	2.38 ± 1.30	67% (38)	2.74 ± 0.94	0.12
Muscle actions	100% (8)	3.38 ± 0.52	93% (53)	3.39 ± 0.62	0.40
Muscle innervation	100% (8)	3.25 ± 0.46	98% (56)	3.65 ± 0.52	0.74
Nerve plexuses	88% (7)	3.13 ± 0.64	98% (56)	3.65 ± 0.52	0.11
Peripheral motor & sensory distribution	88% (7)	3.00 ± 0.53	95% (54)	3.47 ± 0.60	0.44
Vasculature	75% (6)	2.50 ± 1.07	91% (52)	3.33 ± 0.64	0.18
Clinical correlates	75% (6)	3.13 ± 0.83	96% (55)	3.42 ± 0.57	0.02*

^a^ Data for Canadian medical programs were collected on a follow‐up survey (*n* = 8). (*p ≤ 0.05), (**p ≤ 0.01).

Finally, 14 Canadian (100%) and 53 American (94.6%—*note: two respondents failed to complete this question*) programs reported using some type of formative assessment to provide students with feedback about their MSK anatomy learning and knowledge retention—this most commonly took the form of ongoing in‐course assessment (nine Canadian—64%, 48 American—86%). The vast majority of programs (13 Canadian—93%, 53 American—98%; *note: two American programs failed to complete this question*) also reported using some type of summative assessment to evaluate students' knowledge and comprehension. This most commonly took the form of end‐of‐course written exams (10 Canadian—71%, 42 American—81%) and end‐of‐course lab‐based examinations (eight Canadian—57%, 37 American—71%). Additionally, all but one Canadian (93%) and all American (100%) programs reported using end‐of‐course evaluations to gauge student satisfaction with the preclinical MSK anatomy curricula.

## 4 Discussion

To our knowledge, this is the first investigation to report curricular data specific to the preclinical MSK anatomy education across nationally accredited allopathic medical programs in both Canada and the United States. The results build on data recently published by Wang et al. (2021) and Peeler (2024), and suggest that despite rigorous accreditation standards for North American medical programs, there is significant heterogeneity (Wang et al. 2021; Peeler et al. 2024). They provide an important “snapshot” in time that documents the current learning environment, and serve as a baseline to which MSK anatomy curricula in other jurisdictions can be compared both now and in the future. Finally, the results are also believed to be generalizable to all nationally accredited undergraduate allopathic medical programs governed by the Liaison Committee on Medical Education (LCME).

The survey data indicated that the total number of curricular hours varied widely across Canadian (mean: 29.8 h ± 13.7, range 12–60) and American (mean: 50.8 h ± 46.2, range 2–280) medical programs, most of them integrating their MSK anatomy instruction into a larger clinical MSK course. These findings mirror those by Wang et al. (2021), who reported little consistency in the total number of MSK anatomy hours (range 4–50) and methods of delivery used by the 11 California‐based medical programs (Wang et al. 2021). These large variations in contact hours also reflect data previously included in the MSK medical education literature (Grunfeld et al. 2012; Sabesan et al. 2018; Drake et al. 2009; Rockarts et al. 2020; Orchard et al. 2024; Wang et al. 2021; Peeler et al. 2024), and indicate that despite the significant burden and large socioeconomic cost associated with MSK injuries/diseases, opportunities for physician learning in MSK anatomy continue to be underrepresented in the preclinical curricula of most North American medical programs (Pinney and Regan 2001; Wang et al. 2021; Peeler et al. 2024). While consensus among educators about the number of hours required for physician trainees to learn MSK anatomy effectively seems unlikely, our previous research, which examined the efficacy of MSK anatomy teaching at a nationally accredited medical program, indicated that a 30‐h preclinical MSK anatomy course was associated with high rates of student satisfaction and MSK anatomy knowledge retention (Peeler 2022, 2024; Orchard et al. 2024). Interestingly, in the present study, 64% of programs (seven Canadian—50%; 39 American—67%) included a minimum of 30 h of MSK anatomy instruction.

The survey results showed that nearly all programs (71 of 72%–99%) used some form of cadaveric‐based instruction to support student learning, radiological correlates (51 of 72%–71%) and surface anatomy/palpation (68 of 72%–94%) also being included in most curricula. These findings align with data from our previous investigation, which indicated that students prefer curricula that use cadaveric‐based materials (dissection and/or prosection) and radiological imaging to support learning (Whelan et al. 2018). These modes of delivery offer several advantages, including the ability to visualize a structure in vivo and gain a greater appreciation and understanding of the spatial relationships among anatomical structures (Estai and Bunt 2016). They provide inexperienced learners with opportunities to view/learn about a range of pathologies while directly comparing them to normal anatomy (Gunderman and Wilson 2005). Most importantly, they allow students to develop skills that are applicable to a real‐world setting—using surface anatomy/palpation during a physical examination; interpreting radiology images; or demonstrating detailed knowledge of the spatial relationships of structures when performing common medical procedures (e.g., joint injection/aspiration) (Gradl‐Dietsch et al. 2016).

Having said this, the total hours devoted to cadaveric‐based instruction and the detail with which core topics were covered within each medical curriculum were highly variable. This finding is likely to be attributable to the type of instruction that was offered—programs that used dissection‐based instruction allocated approximately twice the instructional time of those who used prosection. Additionally, no core topics in MSK anatomy were “covered” or “covered in detail” by all Canadian and American programs (Pinney and Regan 2001). Although all Canadian programs taught four of the 17 topics (Bones of the axial and appendicular skeleton; Bony features/vocabulary; Muscle actions; and Muscle innervations), many of the others were not covered. This finding illustrates that inadequacies persist within the MSK anatomy curricula of North American medical programs (Wang et al. 2021; Peeler et al. 2024). Although previous literature has outlined similarities in core MSK anatomy topics required for preclinical and postgraduate training, the results indicate a need for greater MSK anatomy training during preclinical and postgraduate years to provide physicians with adequate anatomical knowledge to prepare them for clinical practice (Lisk et al. 2014; Hankin et al. 2023; Harmon et al. 2024). Despite rigorous accreditation standards, and ongoing initiatives designed to improve physician training in MSK medicine, these results suggest an ongoing need for programs to identify and adopt a standardized set of MSK anatomy topics that would be taught by all accredited programs.

The survey data also indicated that programs continue to expand the modes of delivery that are used to teach MSK anatomy content to preclinical students. While most respondents (64 of 72%–89%) indicated that their program still used a lecture/lab format, data also suggested that other modes of instruction have become commonplace within curricula, creating a more diverse learning environment. This includes lab‐based activities that do not use cadaveric materials—more than 70% of programs use other learning aids (muscle models, visualization software, etc.) within their labs; self‐study/asynchronous learning that allows students the freedom to learn at their own pace in an “on‐demand” manner; case‐based tutorials that promote a team approach to decision‐making; and online learning sessions delivered synchronously that take advantage of video‐conference platforms (e.g., Zoom or Microsoft Teams). These results are consistent with recent reports within the literature (Harkins et al. 2023; Wang et al. 2021; Peeler et al. 2024; Estai and Bunt 2016; Gunderman and Wilson 2005; Gradl‐Dietsch et al. 2016) and are further evidence that a “one size fits all” approach no longer exists across programs. Instead, most programs use a multi‐model approach, which relies upon didactic lectures and cadaveric‐based instruction as pillars for learning around which other modes of instruction revolve.

The results suggested several trends in the learning resources that are provided to students. Less than half of the Canadian programs used a required anatomy textbook/atlas to support learning. Instead, most of them used an institution‐specific notes package. In contrast, most American programs used a “required” anatomy textbook (Moore's Clinically Oriented Anatomy—14 (25%); Gray's Anatomy for Medical students—nine (16%)) or atlas (Netter Atlas of Human Anatomy—23 (43%); Grant's Atlas of Human Anatomy—11 (20%)) to support learning. Interestingly, this contradicts recently published data that indicated a growing trend toward computer‐based “apps” as a primary learning resource (Harkins et al. 2023). In total, 63 of the programs (88%) indicated that they provided their students with a curriculum‐specific notes package, with few programs continuing to require their students to create their own notes. These findings can probably be attributed to a host of factors including (but not limited to): (Fontánez et al. 2021) The financial burden associated with purchasing the most recent edition of an anatomy textbook/atlas; (Weinstein et al. 2014) Student preference for easily accessible/interactive low‐cost computer‐based apps; and (Anderson and Althausen 2016) Technological advances that have made the development of curriculum‐specific notes packages simple and cost‐effective.

Each program reported using various methods of formative and summative assessment to evaluate students' knowledge of MSK anatomy. Previous research has shown that formative assessment positively influences a student's performance on end‐of‐term examinations when the goal of feedback is to assist the student to self‐assess their knowledge and take action to address any gaps (Mitra and Barua 2015). Our data indicated that the most common formative assessments provided were “*ongoing in course practice questions, quizzes, and tests*” (57/70%–81%). Despite evidence suggesting that performance on high stakes end‐of‐course examinations is confounded by a host of variables and can therefore lack validity (Van der Vleuten 2000), the summative assessments most commonly used to gauge learning were “*end‐of‐course written exams*” (52/68%–74%) and “*end‐of course cadaveric‐based lab exams*” (45/68%–64%). While justification for the continued use of these examinations is beyond the scope of this investigation, it is likely that these modes of summative assessment persist because of factors related to assessment bias (e.g., the one size fits all approach), program history (e.g., “that's the way we have always done it”), and challenges that arise from trying to fit multiple modes of evaluation into a time‐constricted curriculum.

Finally, programs were consistent in their use of end‐of‐course student surveys (quantitative and qualitative) for evaluating the preclinical MSK anatomy learning environment, only one program indicating that it did not collect end‐of‐course data. This finding can probably be attributed to the fact that both Canadian and American accreditation processes place significant emphasis on the role of end‐of‐course evaluations in ensuring that a “student voice” is heard when decisions regarding curricular design and implementation are made.

This study is not without limitations. Response bias among survey respondents is likely, especially in the absence of a “gold standard” curriculum. Beyond this, the organization, structure, and curricular details differed widely among programs, and in some instances, a single individual in an institution could have found it difficult to provide complete/detailed responses to all the questions. For these reasons, it is also possible that we misinterpreted a program's specific response to an individual question. Finally, a detailed comparison of MSK anatomy course learning objectives and course content among programs was beyond the scope of this investigation.

## 5 Conclusions

The purpose of this study was to document the current state of preclinical MSK anatomy education across nationally accredited medical programs in North America. It builds on the recent publications by Wang et al. (2021) and Peeler et al. (2024) about MSK medicine education, and is the first to report curricular data specific to preclinical MSK anatomy education in both Canadian and American medical schools (Wang et al. 2021; Peeler et al. 2024). The data confirm a high degree of variability among accredited medical programs in terms of MSK anatomy course contact hours, curricular content, modes of instruction, learning resources, and assessment methods used to evaluate MSK anatomy learning and knowledge retention. The results represent an important “snapshot in time” and illustrate a need for nationally accredited programs to provide more consistent and comprehensive preclinical training in MSK anatomy. The results constitute a baseline against which MSK anatomy curricula of other accredited programs can be compared both now and in the future. Finally, it is hoped that these results will provide a first step towards the creation of medical education guidelines for MSK anatomy education. Future studies should be directed at establishing an educational “gold standard” for preclinical MSK anatomy education within nationally accredited allopathic medical programs, and at investigating the long‐term effect of the preclinical MSK anatomy learning environment on postgraduate physician knowledge retention and clinical confidence.

## Ethics Statement

Institutional ethics approval was received for this research.

## Consent

All respondents consented to the release of their program's MSK anatomy curriculum information.

## Supporting information


**Data S1.** Supporting Information.
