# Supplementary material for: Musculoskeletal Anatomy Education: A Survey of North American Medical Programs
Source: Clin Anat. 2025 Apr 28;38(5):568–75. doi: 10.1002/ca.24282 (PMC12163103; doi:10.1002/ca.24282)
Supplement: Supplementary file 1 — Data S1. Supporting Information. [file CA-38-568-s001.docx]

**Musculoskeletal Anatomy Education Survey**

**(***Adapted from Wang et al, 2021 and Peeler et al, 2024***)**

*** Answer required**

*1. In which province / state is your accredited program located (please list):

__________________________

*2. Approximately, how many medical students are admitted to your AAMC/AFMC accredited program on an annual basis?

__________________________

*3. Does your medical program require students to complete a pre-requisite human anatomy course prior to admission to medicine?

- Yes
- No

*4. Does your accredited program teach MSK anatomy as part of its accredited curriculum?

- Yes, it is delivered as a standalone MSK anatomy course.
- Yes, it is delivered as part of a larger systems-based anatomy course that includes other anatomy topics (eg. embryology, histology, cardio-respiratory, neuroscience, gastrointestinal, etc.).
- Yes, it is delivered as part of a larger integrated course that includes clinical topics such as MSK injury epidemiology; MSK injury recognition, management & prognosis; and/or clinical skills
- No, our program does not teach MSK anatomy as part of its accredited curriculum.

If yes, when is the MSK anatomy course/curriculum delivered? *Please check all that apply*.

- 1^st^ year
- 2^nd^ year
- 3^rd^ year
- 4^th^ year

*5. Approximately how much time would students who are enrolled in your accredited program spend learning MSK anatomy over the duration of their medical degree?

________ (*total hours)*

*6. Does your MSK anatomy curriculum involve the use of any of the following teaching and learning activities? *Please check all that apply.*

- Lab-based activities using cadaveric materials
- Lab-based activities using other learning materials (eg. plastic models, images, etc)
- Didactic lectures / review sessions
- Case-based learning
- Online or computer-based learning (synchronous)
- Self-study / asynchronous learning
- Other (please specify): ______________________________________________________________________________________________________________________________________________________________________

*7. If the lab-based activities involve the use of cadaveric materials, what type of activities are used and how many hours are spent on each activity?

- Dissection-based - ______ (*hours)*
- Prosection-based - ______ (*hours)*
- Both – dissection - ______ (*hours*); prosection - ______ (*hours*)

*8. For each of the following MSK anatomy topics, please note the extent to which they are covered within your program’s MSK anatomy course / curriculum

Not Addressed May be Covered Covered Minimally Covered Covered in Detail

(but not assured)

Embryology of MSK system 🞎 🞎 🞎 🞎 🞎

Histology of the MSK system 🞎 🞎 🞎 🞎 🞎

Anatomical terminology 🞎 🞎 🞎 🞎 🞎

Bones (axial & appendicular skeleton) 🞎 🞎 🞎 🞎 🞎

Bony features 🞎 🞎 🞎 🞎 🞎

Joints 🞎 🞎 🞎 🞎 🞎

Joint function & range of motion 🞎 🞎 🞎 🞎 🞎

Ligaments & supporting structures 🞎 🞎 🞎 🞎 🞎

Ligamentous attachments 🞎 🞎 🞎 🞎 🞎

Muscles 🞎 🞎 🞎 🞎 🞎

Muscular attachments 🞎 🞎 🞎 🞎 🞎

Muscle actions 🞎 🞎 🞎 🞎 🞎

Muscle innervation 🞎 🞎 🞎 🞎 🞎

Nerve plexuses 🞎 🞎 🞎 🞎 🞎

Peripheral / terminal nerves 🞎 🞎 🞎 🞎 🞎

Vessels – main 🞎 🞎 🞎 🞎 🞎

Vessels – peripheral 🞎 🞎 🞎 🞎 🞎

Clinical correlates 🞎 🞎 🞎 🞎 🞎

*9. Which of the following types of educators are involved in the instruction of the MSK anatomy curriculum within your medical program? *Please select all that apply*.

- PhD trained faculty
- Non-PhD trained faculty who hold a clinical designation
- Clinicians from the community (i.e. professionals who see patients regularly)
- Graduate students
- Other (*please specify*): ___________________________________________

*10. What would the typical instructor-to-student ratio be for lab-based activities that are included in the MSK anatomy curriculum within your medical program? *One instructor per ________ students*.

__________________________

*11. Which of the following learning resources does your MSK anatomy course / curriculum utilize to support student learning? *Please check all that apply.*

- Standardized lecture or notes package
- Standardized lab manual or notes package
- Required / recommended anatomy atlas

*If so, which one: ______________________________*

- Required / recommended anatomy textbook

*If so, which one: ______________________________*

- Computer-based resources (apps / learning software)

*If so, which one: ______________________________*

- Students are required to develop their own study notes
- Other (please specify): ______________________________________________________________________________________________________________________________________________________________________

*12. Is MSK surface landmarking or palpation of anatomical structures included as part of your MSK anatomy course / curriculum?

- Yes
- No

If yes, to what extent does your MSK anatomy course / curriculum include MSK surface landmarking or palpation (e.g. number of curricular hours, modes of delivery, etc.)? *Please describe.*

__________________________________________________________________________________________________________________________________________________________________________________________________________________________________________________________________

*13. Are radiological correlates (X-ray, CT, MRI, Ultrasound) taught as part of the MSK anatomy course / curriculum

- Yes
- No

If yes, to what extent does your MSK anatomy course / curriculum include radiological correlates (e.g. number of curricular hours, modes of delivery, etc.)? *Please describe.*

__________________________________________________________________________________________________________________________________________________________________________________________________________________________________________________________________

*14. What methods of ***formative evaluation*** are used to provide students with feedback regarding their MSK anatomy knowledge in your medical program? *Please check all that apply.*

- No methods of formative evaluation are used in our MSK anatomy course /curriculum
- Ongoing in-course practice questions / quizzes / tests
- Mid-course lab practice exam
- Mid-course MCQ written exam
- Mid-course short/long answer written exam
- End-of-course lab practice exam
- End-of-course MCQ written practice exam
- End-of-course short/long answer written exam
- Other (please specify): ______________________________________________________________________________________________________________________________________________________________________

*15. What methods of ***summative evaluation*** are used to evaluate MSK anatomy knowledge in your medical program? *Please check all that apply.*

- No methods of summative evaluation are used in our MSK anatomy course / curriculum
- Mid-course lab exam using cadaveric materials
- Mid-course lab exam using digital images
- Mid-course written exam (MCQ, short or long answers)
- End-of-course gross lab exam using cadaveric materials
- End-of-course lab exam using digital images
- End-of-course written exam (MCQ, short or long answers)
- Ongoing in-course evaluation using multiple quizzes / tests
- Other (please specify): ______________________________________________________________________________________________________________________________________________________________________

*16.  Are instructors satisfied with the mode(s) of assessment used to evaluate student’s MSK anatomy knowledge?

- Yes
- No

If no, please describe why?

__________________________________________________________________________________________________________________________________________________________________________________________________________________________________________________________________

*17.  Upon completion of your program's MSK anatomy course(s)/curriculum, how prepared are students to apply their MSK anatomy knowledge in a clinically relevant and confident manner when they are subsequently asked to approach/triage basic MSK clinical conditions?

- 1
- 2
- 3
- 4
- 5

*18. Does your MSK anatomy course ask students to complete end-of-course evaluations?

*Please check all that apply.*

- No end-of-course student feedback is collected.
- Yes, quantitative feedback is collected using a Likert scale.
- Yes, qualitative feedback is collected using narrative comments.
- Other (please specify): ______________________________________________________________________________________________________________________________________________________________________

*19. Does your MSK anatomy course incorporate equity, diversity, inclusiveness, and decolonization (EDID) principles?

*Please check all that apply.*

- Racial diversity is represented in anatomical images used.
- Body diversity is represented in anatomical images used.
- Eponyms are avoided where possible.
- Gender inclusive terminology is incorporated when discussing MSK related sex differences.
- Other (please specify):

_______________________________________________________________________________

_______________________________________________________________________________

***Optional – If you would like to provide your contact information, please do so below:***

***Name: ________________________________________***

***Institution: ________________________________________***

***Email: ________________________________________***
